# mRNA extracted from frozen buffy coat samples stored long term in tubes with no RNA preservative shows promise for downstream sequencing analyses

**DOI:** 10.1371/journal.pone.0318834

**Published:** 2025-03-19

**Authors:** Erik Bovinder Ylitalo, Linda Vidman, Sophia Harlid, Bethany Van Guelpen

**Affiliations:** 1 Department of Diagnostics and Intervention, Oncology, Umeå University, Umeå, Sweden; 2 Wallenberg Centre for Molecular Medicine, Umeå University, Umeå, Sweden; Saga University, JAPAN

## Abstract

Transcriptomics is an important OMICs method that is often unavailable in biobank research. Frozen blood samples are routinely collected and stored in medical biobanks, but transcriptional studies have been limited due to technical difficulties of extracting high-quality RNA from blood frozen in standard tubes (without RNA preservatives). We aimed to determine whether biobanked buffy coat samples stored at -80°C for up to 23 years could be successfully used for mRNA sequencing. We used a CryoXtract CXT 350 to remove frozen sample cores, which were immersed in RNA preservative during thawing prior to RNA extraction. RNA sequencing was then performed on extractions from pooled samples as well as from 23 buffy coat samples from prospective colorectal cancer cases and 23 matched controls included in the population-based, prospective Northern Sweden Health and Disease Study (NSHDS). For all samples, two library preparation methods were used (Illumina TruSeq Stranded mRNA poly-A selection and Illumina Stranded Total RNA with Ribo-Zero Globin). RNA yields of over 1 µg were obtained from the majority of NSHDS samples (mean = 2.57 µg), and over 92% of samples had RIN values of ≥ 6, indicating suitability for downstream analyses. In conclusion, we developed a method for successfully extracting and sequencing high-quality mRNA from frozen buffy coat samples stored long term in tubes with no RNA preservative.

## 1. Introduction

Human blood samples are routinely collected and stored in medical biobanks across the world. Many such biobanks are established within prospective cohorts for use in future medical research. Over time, some cohort participants will be diagnosed with various diseases and there are numerous examples of prediagnostic biobanked blood samples being used to gain knowledge of etiology, risk or, increasingly, for the identification of markers for early detection of disease [1–5].

One previously underused blood biomarker is messenger RNA (mRNA), which can be extracted from the white blood cell fraction commonly known as buffy coat. Given the critical role of the immune response and inflammation in the development of many chronic diseases, including cardiometabolic diseases and cancer, transcriptomic alterations in circulating immune cells represent a potential novel early-detection biomarker source. Differences in immunological gene expression in the years prior to diagnosis of a disease could also reflect the physiological environment promoting disease development, which could be used to gain new etiological insights or to develop prevention strategies based on risk stratification.

Historically, transcription studies on frozen blood samples from biobanks have been limited due to technical difficulties. Samples are generally collected in tubes without RNA preservative, which can result in heavy degradation rendering the samples unsuitable for downstream use in advanced transcriptomic analyses, such as microarrays and RNA sequencing. However, using the right tools, it is possible to isolate RNA of acceptable quality from frozen blood collected in standard tubes [6–8]. This has been demonstrated in samples from future lymphoma patients and matched cancer-free controls, in which RNA from buffy coat samples collected up to 17 years prior to diagnosis had sufficient quality for microarray analysis [9]. In that study, differentially expressed genes for chronic lymphoma leukemia were identified, which were consistent in two independent cohorts and later described as having substantial overlap with expression profiles in clinical samples [10]. Assuming a reasonable collection-to-storage time (less than 8 hours) [8], the key point to limit RNA degradation is to inhibit RNase activity during the thawing process, for example by thawing the samples in the presence of an RNA preservative. Previously, this entailed a labor-intensive process of manual removal of frozen samples from tubes, division with a clean scalpel and thawing in an RNA preservative.

One of the cohorts used in the previous studies on lymphoma was the population-based Northern Sweden Health and Disease Study (NSHDS), which includes more than 140 000 participants and over 275 000 biobanked blood samples. Our research group uses the NSHDS cohort for biomarker studies of colorectal cancer etiology, risk prediction and early detection. CRC is the third most common type of cancer in both men and women [11]. When detected early up to 90% of patients will be cured [12], whereas the 5-year survival for patients with metastatic CRC drops to less than 30% [13] despite improvements in therapies. Prevention strategies, including public health approaches and general screening programs, are therefore central to reduce CRC mortality. To our knowledge, blood-based gene expression profiling has not been explored in a prediagnostic setting for CRC. However, for a large-scale study, a more high-throughput methodology for extracting RNA of sufficient quality and quantity would be required.

In this study, we investigated the potential of using frozen blood samples from the NSHDS cohort for RNA sequencing. First, we established a cryoextraction protocol capable of isolating high-quality RNA without thawing the entire sample. We then extracted RNA from 23 prospective colorectal cancer patients and 23 matched controls, from the NSHDS, and performed RNA sequencing using two different library preparation methods.

## 2. Materials and methods

### 2.1. Setting and samples

The blood samples included in this study originate from the large population-based research cohorts in northern Sweden collectively called the Northern Sweden Health and Disease Study (NSHDS), which was initiated in 1985 (first participant 01-10-1986, recruitment ongoing). NSHDS consists of three subcohorts: the Västerbotten intervention programme (VIP), the Mammography Screening Project and the Northern Sweden MONICA study, all previously described [14]. This study used the VIP and MONICA cohorts, which had more stringent and consistent sample handling. From within an existing case-control study of colorectal cancer, nested in the NSHDS, we selected 23 cases and 23 cancer-free control participants matched by cohort, sex, age, year of sampling and fasting status (sample collection dates 25-03-1998 to 28-04-2011, colorectal cancer diagnosis dates 21-05-2004 to 29-03-2016). Participants with a previous cancer diagnosis, other than non-melanoma skin cancer, were excluded. Samples were collected 5-9 years prior to diagnosis, and all samples were previously unthawed. Samples were accessed on 10-02-2021, after which RNA prep was completed within three months.

In order to test and optimize the cryoextraction and RNA extraction method, before using it on valuable samples from the NSHDS biobank, a training set of fully anonymized frozen buffy coat samples (*n* = 25) was obtained from Biobanken Norr (the medical biobank responsible for the NSHDS blood samples). Finally, to investigate the effect of freezing and cryoextraction on RNA quality, Biobanken Norr provided a pooled sample of anonymized fresh buffy coat samples, which we used to compare RNA integrity between RNA extracted from fresh buffy coat, frozen buffy coat and frozen buffy coat that included a cryoextraction step. As part of the anonymization procedure, the number and characteristics of individuals included in the pooled sample were not revealed to the researchers.

The project was approved by the regional ethical review board in northern Sweden (2015/172-31M, 2015/391-32M) and was conducted in accordance with Swedish law and the European General Data Protection Regulation, including use of pseudonymized personal data with no direct identifiers. Written informed consent was obtained for all individual samples.

### 2.2. Blood sample collection, handling and storage

Blood samples provided by participants in the NSHDS are collected in one 10 mL EDTA tube and one 10 mL heparin tube, each of which is divided into fractions (three containing plasma, one containing erythrocytes and one containing buffy coat). No samples are stored in RNA preservative. The collection-to-freezer time is less than one hour, and the samples are placed either directly in -80°C freezers or stored in -20°C freezers for a maximum of one week before transfer to -80°C for long-term storage. Although blood stored in heparin tubes can generate high quality RNA, heparin might also cause technical problems by inhibiting crucial enzymes often used in downstream transcriptomic analysis [15]. To avoid this issue, we used only samples collected in EDTA coated tubes. For samples included in the training set, the collection, handling, and storage procedures were very similar to those routinely used for the NSHDS samples. For the pooled buffy coat sample, a somewhat different collection procedure was used. In brief, blood intended for the pooled sample was collected and fractioned (into plasma, erythrocytes and buffy coat) during the morning and then kept refrigerated until the afternoon when the buffy coat fractions were pooled into a single sample tube and transported on ice to our lab. A portion of the pooled buffy coat sample was then aliquoted into four samples of 150 μL each. Two of these samples were used for immediate RNA extraction (fresh) and two samples were stored at -80°C for nine days after which the RNA was extracted (frozen). The remaining sample volume in the original tube (~400-500 μL) were stored at -80°C for an arbitrary time period of just over a week. Cryoextraction from the original tube was done on day 8 (hereafter referred to as “frozen + cryoextracted”). RNA was extracted from both the frozen and frozen +  cryoextracted samples on day 9.

### 2.3. Cryoextraction

**Fig 1:**
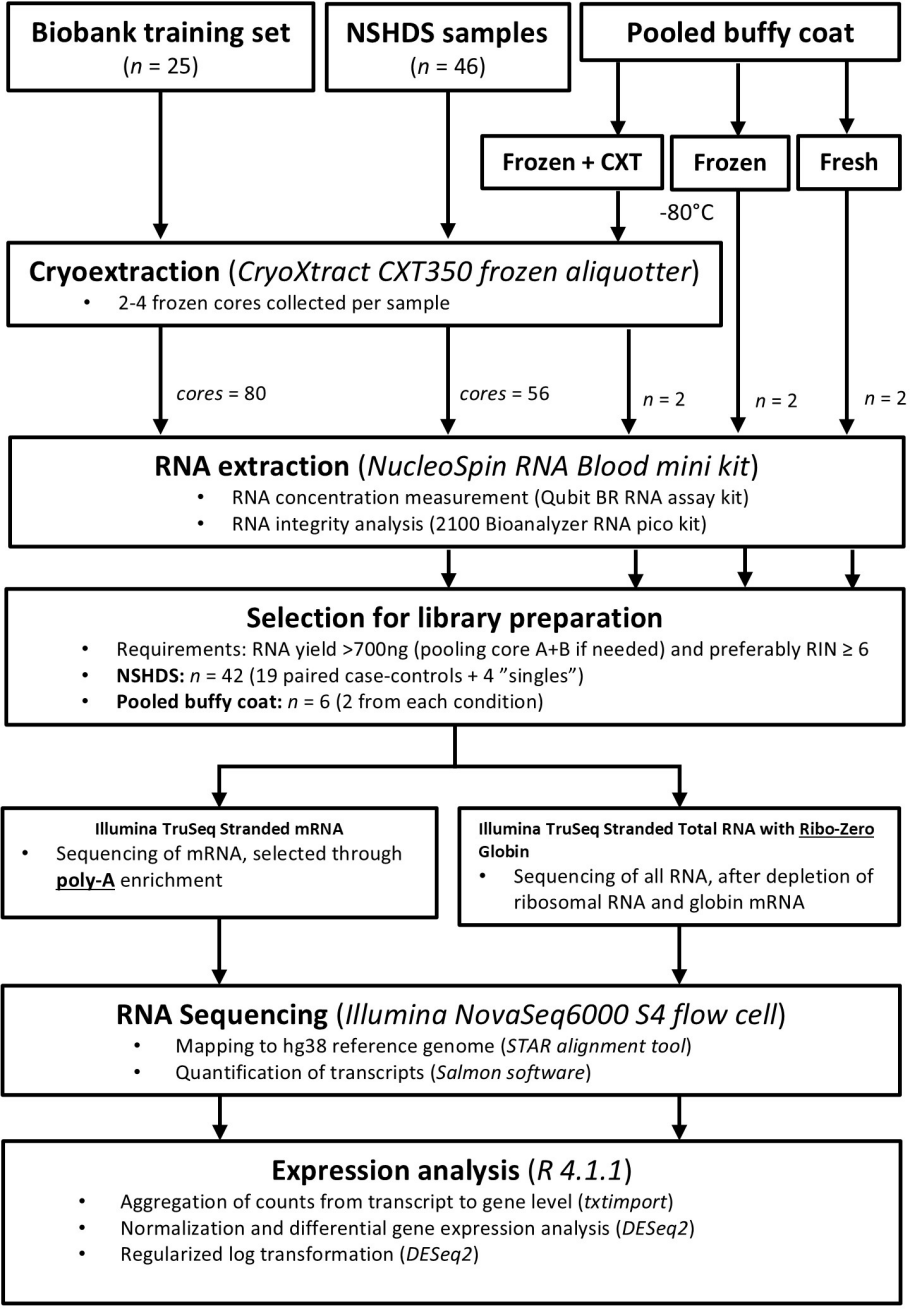
Flowchart depicting the origin of all sequenced samples.

For samples subjected to cryoextraction, the protocol flowchart is shown in Fig 1. A CryoXtract CXT 350 Frozen Aliquotter (Basque Engineering +  Science) was used to collect frozen core aliquots. Samples were kept frozen during the entire process, being transported on dry ice between the freezer and workspace, by mounting the sample tubes in a metal chilling fixture submersed in liquid nitrogen during cryoextraction, and by using pre-chilled, single-use, nuclease-free probes (3.0 mm diameter, 57 mm long) in the cryoextractor. In accordance with our formal agreement with Biobanken Norr, two frozen cores were collected from each NSHDS sample tube: core A and B. For the training samples, up to four cores, cores A-D, were extracted from each sample tube. The exact number of cores per sample was the maximum practically possible, depending on the total volume and orientation of the sample in the tube. For the frozen +  cryoextracted pooled samples, two cores were collected. Cores were immediately transferred to individual Eppendorf tubes and kept frozen using liquid nitrogen and dry ice prior to transfer to a -80°C freezer for storage until RNA extraction. For the pooled sample (frozen +  cryoextracted) the cores were stored for one day, to match the nine-day total storage time of the non-cryoextracted (frozen) samples. After collecting the frozen cores, the original frozen NSHDS sample tubes were returned to the biobank freezers with a fully uninterrupted cold chain.

### 2.4. RNA extraction

RNA was extracted using the NucleoSpin RNA Blood kit (Macherey-Nagel). For the fresh and frozen aliquots from the pooled sample, the manufacturer’s suggested standard protocols were followed. For cryoextracted samples, one RNA extraction was performed for each sample core. All cores were kept frozen on dry ice before initiating the thawing process by adding 200 µ L of RNA lysis buffer directly into the sample tubes containing the frozen core. When thawed, the volume was measured using a pipette and samples were diluted with RNase-free water to a total volume of 400 µ L before addition of 5 µ L Proteinase K followed by incubation with vigorous shaking for 15 min at room temperature. The lysates were mixed with 200 μL of 70% ethanol, transferred to a NucleoSpin RNA Blood column, washed with 350 μL MDB, and 95 μL of rDNase was added to the spin column. After a 15 min incubation at room temperature the column was washed using buffers RB2 and RB3. The RNA was eluted in a two-step process using 40 µ L RNase-free water for the first elution, and the eluate was added once more to the column for the second elution.

RNA quantification was performed using the Qubit BR RNA assay kit (Thermo Fisher Scientfic), and RNA integrity was evaluated using the Eukaryote Total RNA Pico assay on the Agilent 2100 Bioanalyzer (Agilent Technologies). An RNA integrity number (RIN) [16] of at least 6 was considered a minimum level of quality to test in downstream sequencing analyses.

### 2.5. RNA sequencing

RNA sequencing, including cDNA library preparation, was performed at the National Genomic Infrastructure (NGI) Sweden (SciLifeLab). All samples sent for RNA sequencing were processed using two different methods for cDNA library preparation, Illumina TruSeq Stranded mRNA poly-A selection (200 ng RNA) and Illumina Stranded Total RNA with Ribo-Zero Globin (500 ng RNA), followed by sequencing on a NovaSeq6000 S4 flow cell (Illumina).

After sequencing, mapping of reads to the human reference genome (hg38) was performed using STAR alignment tool [17] and transcripts were quantified using Salmon [18]. The RNA sequencing analysis pipeline is described in more detail elsewhere [19].

### 2.6. Gene expression profiles

Aggregation of counts from transcript to gene level was made by *tximport* [20]. Differential gene expression analysis was performed on count data using DESeq from R-package *DESeq2 *[21] with a default cutoff of 0.1 for false discovery rate. For all other analyses, the data were transformed using regularized logarithm (rlog), which normalizes the data with respect to library size and transforms the data to the log2 scale.

### 2.7. Statistical analysis

All analyzes were performed in software R version 4.1.1 [22]. P-values below 0.05 were considered significant. Correlation between the two library preparation methods were assessed by comparing gene expression profiles after removing the twenty percent most low-expressed features (among non-zero features). Spearman correlation was calculated for each individual based on all features that overlapped between the poly-A and Ribo-Zero data and then averaged over the samples. This value was compared to the average correlation between all samples within the same dataset. The analysis was repeated including only the overlapping features after selecting the 1000 genes with highest standard deviation in both datasets separately.

Spearman correlation between the expression of 23 housekeeping genes [23] and RIN value was calculated for both poly-A and Ribo-Zero data. Spearman correlation was also used for calculating correlation between RIN values, time in freezer and number of uniquely mapped reads. Wilcoxon rank sum tests were used for testing differences between RIN values and RNA yields.

Classification of sex was performed using random forest analysis based on both all non-zero features and top 1000 features with highest standard deviation. The analysis was done with the r*andomForest* package with parameters Ntree = 3000 and Mtry = 100.

## 3. Results

### 3.1. RNA quantity and quality

**Fig 2:**
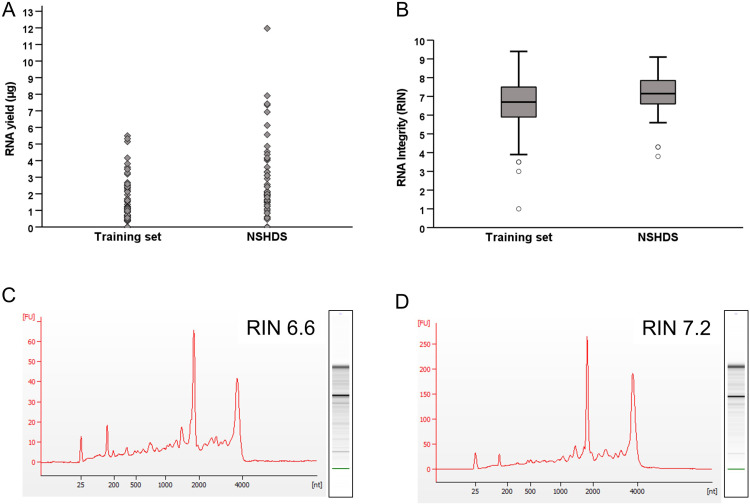
(A) Individual RNA yields from the training samples (n = 80) and the NSHDS samples (n = 56) (total μg from each sample). (B) Variations in RIN values from the training set and the NSHDS samples. (C and D) RIN curves of an individual sample within each sample group which are also representative of the mean RIN values for the training set (C) and NSHDS samples (D).

The first aim of this study was to establish an RNA extraction protocol capable of isolating RNA of sufficient quantity and quality for use in whole-genome transcriptomic analyses such as microarrays and RNA seq. To accomplish this, we extracted RNA from a set of 25 frozen buffy coat training samples collected, handled and stored in a manner similar to that of the NSHDS samples. We were able to cryoextract a total of 80 cores from these samples, yielding a mean of 1.25 ±  1.36 µg (mean ±  SD) RNA per core, ranging from less than 35 ng (RNA concentration below the detection level of 1 ng/ µ L in a total sample volume of 35 µ L) to 5.5 µg (Table 1, Fig 2A and S1 Table). Results from the RNA quality assessment showed a mean RIN value of 6.6 ±  1.4 (range 1.0–9.4). In total, 77.5% of the samples had RIN values of 6 or above (Table 1, Fig 2B and C and S1 Table).

**Table 1:** Integrity and yield of RNA extracted from frozen buffy coat samples.

Cohort	n*	RNA yield (µg)	RNA integrity (RIN)	No. of samples with RIN ≥ 6
Training set	80	1.25 ± 1.36 (0**–5.50)	6.6 ± 1.4 (1.0–9.4)	62 (77.5%)
NSHDS	56	2.57 ± 2.40 (0**–12.0)	7.2 ± 1.1 (3.8–9.1)	52 (92.9%)

Data are presented as mean ±  standard deviation. Numbers in parentheses represent the data range (RNA integrity and RNA yield) or the percentage of the total samples (samples with RIN ≥ 6). *n refers to number of extracted cores. ** RNA yield is based on a sample eluate of 35 µ L. RNA concentration was measured using Qubit BR RNA assay (ThermoFisher Scientific) and measurements below the detection range (1 – 1000 ng/ µ L) were set as 0.

After successfully establishing the RNA extraction protocol, we used the protocol to extract RNA from a set of 46 buffy coat samples from NSHDS participants (23 CRC case-control pairs with samples collected prior to diagnosis). A flow chart of sample selection is shown in Fig 1 and participant characteristics in Table 2. Using the CryoXtract we removed two cores from each sample tube (core A and B) and extracted RNA from core A for each sample. Core B was used as a back-up sample, and we only extracted RNA from these cores if the yield and/or RNA integrity from core A was considered insufficient. In total, we extracted RNA from 56 cores (of which 10 from core B), with an average yield of 2.57 ±  2.40 µg RNA, ranging from <  35 ng to 12 µg (Table 1 and Fig 2A). The mean RIN value was 7.2 ±  1.1 (range: 3.8–9.1), and 92.9% had a RIN ≥  6 (Table 1 and Fig 2A, D).

**Table 2:** Properties of NSHDS samples selected for RNA sequencing.

	Cases	Controls	Total
No. of samples extracted	19	23	42
Age (years)	58.9 ± 5.5 (49.5-70.1)	59.2 ± 4.9 (50.0-69.9)	59.1 ± 5.5 (49.5-70.1)
Sex (male/ female)	6/13	7/16	13/ 29
RNA yield (µg)	3.26 ± 2.00 (1.35 – 7.91)	3.46 ± 2.57 (0.83 – 11.97)	3.37 ± 2.31 (0.83 – 11.97)
RNA integrity (RIN)	7.3 ± 0.7 (6.2-8.6)	7.2 ± 0.9 (5.9-9.0)	7.3 ± 0.8 (5.9 – 9.0)
No of samples with RIN ≥ 6	19 (100%)	22 (95.7%)	41 (97.6%)

Data are presented as mean ±  standard deviation. Numbers in parentheses represent the data range (age, RNA integrity and RNA yield) or the percentage of the total samples (samples with RIN ≥  6).

No significant difference was seen in RIN or RNA yield for cases compared to controls (p = 0.569 and p = 0.940). RIN values had a positive correlation with time in freezer (r = 0.451, p = 0.003). After pooling and excluding any samples that did not meet the minimum requirements regarding RNA quantity ( ≥ 700 ng) or quality (RIN 6 or above), 42 samples remained (19 case-control pairs and 4 control samples). Of these 42 samples, 41 (97.6%), had RIN ≥  6, with the last sample falling just below our predetermined cut-off with a RIN =  5.9. All 42 samples were sequenced using two library preparation methods (Illumina TruSeq Stranded mRNA poly-A selection and Illumina Stranded Total RNA with Ribo-Zero Globin), hereafter referred to as poly-A and Ribo-Zero.

**Fig 3:**
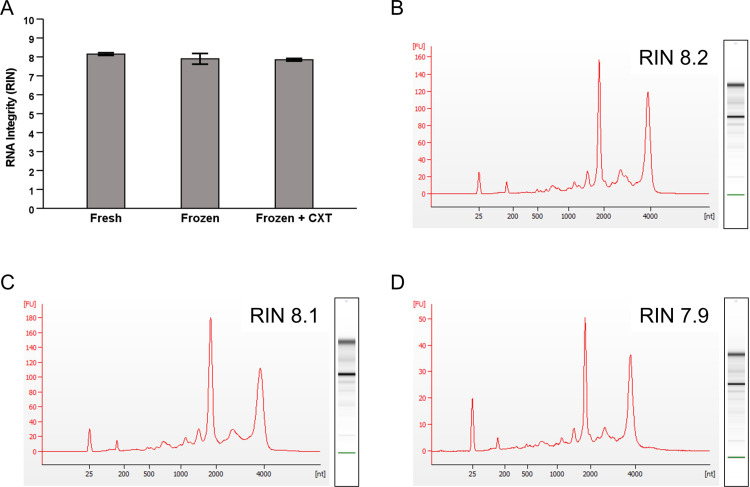
(A) RIN values of RNA extracted from pooled buffy coat after different storage and handling conditions. **(B-D)** **Max RIN curves for fresh** **(B), frozen (C) and frozen +  cryoextracted (D) pooled buffy coat.**

To identify any major effect of the cryoextraction process on RNA sample quality, we also extracted RNA from fresh, frozen and frozen +  cryoextracted aliquots all originating from a single pooled buffy coat sample. The results from the RNA integrity assessment showed RIN values of 8.2 and 8.1 for the fresh aliquots, 8.1 and 7.7 for the frozen aliquots and 7.9 and 7.8 for the frozen +  cryoextracted aliquots (Fig 3).

### 3.2. RNA sequencing

The total number of million reads for each sample varied between 15.5 and 71.1 for poly-A data and between 0.3 and 38.7 for Ribo-Zero. Correlations between number of uniquely mapped reads and RIN value were 0.301 (p = 0.037) and -0.006 (p = 0.632) for poly-A and Ribo-Zero data respectively. Quality scores for each sample, generated through multiQC are presented in S2 Fig and S3 Fig. Three samples in the Ribo-Zero dataset failed the STAR minimum mapped reads check and were therefore removed from all downstream analyses.

### 3.3. Gene expression profiles

The number of detected features (total read count >  0 for at least one sample) were 30 493 for poly-A, and 34 675 for Ribo-Zero. The overlap of detected expressed genes between the samples from the two methods was 28 735.

To study the similarity of expression profiles generated from the samples prepared using the different library preparation methods, we calculated Spearman correlation between the two expression profiles for each individual based on the 18 521 genes that overlapped after removing the 20% lowest expressed features from each data set. The average correlation based on 39 samples was 0.923. That can be compared to the average correlation between expression profiles of all individuals within the same data set (library preparation method) which was 0.998 for poly-A and 0.997 for Ribo-Zero. The procedure was repeated after excluding two outlier samples identified through manual inspection of heatmaps (S4 Fig and S5 Fig), but the correlation remained the same. The same analysis was also made after including only the 40 genes that overlapped when extracting the 1000 genes with highest standard deviation in each data set. The similarity then increased between Ribo-Zero and poly-A expression profiles to 0.955, whereas the average correlation within poly-A and Ribo-Zero became lower 0.992 and 0.988, respectively.

To assess the overall quality of the data, we classified the samples by sex, generating an out-of-bag (OOB) error of 26.2% for poly-A and 28.2% for Ribo-Zero. It is common to perform some type of feature selection or filtering before performing classification analysis, and the error rate dropped for both methods when including only the top 1000 genes with highest standard deviation, yielding an OOB-error of 0% for both poly-A and Ribo-Zero.

Many researchers are hesitant to use low quality RNA samples for gene expression profiling, with one reason being that if degradation occurs at different rates for different transcripts this can introduce bias. To study how RNA integrity might affect downstream analyses we calculated Spearman correlations between RNA integrity (estimated using RIN values) and gene expression for 23 housekeeping genes. For poly-A, 10 genes had a significant negative correlation, and three genes had a significant positive correlation to RIN value. For Ribo-Zero eight of the 23 genes had a significant positive correlation and five genes had a significant negative correlation with RIN value (Table 3).

**Table 3:** Spearman correlation between gene expression of 23 housekeeping genes and RIN values for data generated using two different library preparation methods. P-values < 0.05 are indicated in bold.

	poly-A		Ribo-Zero	
Gene	Correlation	P-value	Correlation	P-value
*ALAS1*	-0.099	0.532	-0.163	0.322
*TFRC*	0.235	0.134	-0.229	0.161
*SDHA*	**-0.535**	**<0.001**	-0.198	0.226
*ACTB*	**-0.532**	**<0.001**	**-0.487**	**0.002**
*RPLP0*	**-0.411**	**0.007**	**0.574**	**<0.001**
*PGK1*	0.183	0.245	**0.512**	**0.001**
*GAPDH*	-0.275	0.078	**0.529**	**0.001**
*TBP*	0.224	0.153	**-0.390**	**0.014**
*ATP5PB*	-0.147	0.354	**0.792**	**<0.001**
*IPO8*	**0.534**	**<0.001**	0.003	0.985
*RPLP1*	**-0.644**	**<0.001**	-0.051	0.757
*RPL13A*	**-0.660**	**<0.001**	**0.564**	**<0.001**
*UBC*	0.048	0.761	**0.621**	**<0.001**
*G6PD*	0.224	0.154	**-0.502**	**0.001**
*YWHAZ*	**0.378**	**0.014**	0.065	0.696
*HPRT1*	-0.148	0.351	-0.081	0.624
*B2M*	0.012	0.938	**0.699**	**<0.001**
*GUSB*	**-0.398**	**0.009**	0.189	0.249
*RPLP2*	**-0.824**	**<0.001**	-0.285	0.078
*POLR2A*	**0.391**	**0.010**	**-0.789**	**<0.001**
*PPIA*	**-0.366**	**0.017**	**0.704**	**<0.001**
*RPS18*	**-0.597**	**<0.001**	0.295	0.068
*HMBS*	**-0.500**	**0.001**	**-0.327**	**0.042**

### 3.4. Differentially expressed genes

Many studies focus on identifying differentially expressed genes between different groups, for example different treatments or individuals with and without a certain disease. Therefore, it is of high importance that the data is of sufficient quality to identify truly differentially expressed genes. We identified 105 differentially expressed genes between the sexes using poly-A (60 upregulated and 45 downregulated for women vs men) and 66 differentially expressed genes using Ribo-Zero (32 upregulated and 34 down regulated), S6 Table. Comparing future CRC cases to controls, one downregulated gene was found using poly-A data (RNA5-8S5) and none using Ribo-Zero.

## 4. Discussion

In this study we developed and tested a novel method for extracting RNA from biobanked buffy coat samples stored without any RNA preservative. Applying our cryoextraction protocol to a test set of 46 biobanked buffy coat samples, at least 700 ng of RNA was successfully extracted from 43 samples (93.5%). Of these, 41 (95.3%) had RIN values ≥  6 suggesting adequate quality for downstream analysis, and because one sample ended up just below this threshold (RIN =  5.9) we decided to also include this sample to make an even set. The samples were sequenced using two library preparation methods, both of which produced gene expression profiles capable of distinguishing between men and women with high accuracy. Overall, our results indicate that it is indeed possible to obtain RNA of both sufficient quantity and quality for use in downstream analyses, including sequencing.

The cryoextraction protocol has several advantages. Removal of a frozen core allows for thawing completely immersed in stabilizing solution, which should help prevent RNA degradation during thawing. More specifically, we thaw the sample cores directly in RNA lysis buffer, which immediately inactivates any RNases present in the sample and thereby effectively limiting RNA degradation. Cryoextraction also preserves the remainder of the original sample in the frozen state, avoiding a freeze-thaw cycle. An added benefit of the protocol is that it may be suitable for higher throughput studies compared to some of the more labor-intensive protocols described before [8,23]. Once the machine is cooled down to the working temperature of -80°C or colder (~10-15 min), the extraction of one core takes less than five minutes. However, the protocol requires access to a cryoextractor and single-use, nuclease-free coring probes, which may not be easily available. Furthermore, if the sample is not thoroughly mixed prior to cryoextraction, cell composition may vary between cores from the same sample. Although a single frozen core generally provided RNA of sufficient quantity and quality for sequencing, nearly a quarter of the samples required use of the second “backup” core due to a failed first core, and a few samples failed completely due to small total sample volumes. Thus, adequate core number is a key factor in planning studies. RIN differences between frozen aliquots and frozen +  cryoextracted cores from the pooled sample were small, but we abstained from statistical comparison testing and cannot draw strong conclusions due to very small sample sizes. The freezer time of 9 days for the pooled sample experiment was arbitrarily selected, but for short-term storage at -80°C, the exact duration of storage (within the span of days) is expected to have minimal impact on RNA quality.

The RNA extracted using our protocol is of similar quality as reported in previous studies in which RNA has been extracted from frozen blood or buffy coat stored without using RNA preservatives (such as standard EDTA tubes) [6–8,23,24]. At least one previous study also evaluated downstream RNA-sequencing on RNA extracted from frozen whole blood (using the Ribo-Zero library preparation kit), but with somewhat discouraging results [23]. However, they used RNA from whole blood and only evaluated one library preparation method.

We also evaluated two separate library preparation methods, poly-A and Ribo-Zero, for use on our samples. The poly-A method uses oligo (dT) primers to selectively bind to and purify poly-A tailed mRNA from the sample. Ribo-Zero, on the other hand, is used to enrich for mRNA by removing ribosomal RNA (rRNA), using RNase to specifically degrade rRNA, leaving mRNA intact. Both methods have been previously compared using multiple different sample types and setups, with polyA + selection being favored due to better coverage of exons and more accurate expression quantification [25].

In our study, samples prepped using Illumina Stranded Total RNA with Ribo-Zero Globin generated higher numbers of uniquely mapped reads compared to samples prepped using Illumina TruSeq Stranded mRNA poly-A selection. Large deviations from expected GC distribution were also seen for library preparation by poly-A selection, which could indicate sample contamination or highly overrepresented sequences. Since Illumina TruSeq Stranded mRNA poly-A selection targets the poly-A tail, which might be lost or fragmented in degraded samples, this might lead to bias toward the 3’ ends of transcripts or a significant loss of mRNA during the selection process [26]. As a result, poly-A selection may be less suitable than Illumina Stranded Total RNA with Ribo-Zero Globin for sequencing of lower-quality RNA samples. Despite these considerations, it is important to note that both methods could identify transcripts from more than 30 000 genes in our study, of which approximately 29 000 overlapped. In addition, both methods allowed us to use differentially expressed genes to distinguish men from women.

Although many of the samples did not pass the library quality controls, we decided, in consultation with NGI (analyzing lab) to go forward with the sequencing step. Usually, the requirements set by RNA sequencing service providers are strict and designed to minimize risk of failed sequencing runs and ensure high quality sequencing results. Similarly, many studies limit their analyses to samples with RIN values above a certain threshold, to ensure robust gene expression data. Transcript degradation in low quality samples can be non-uniform and cause degradation bias in RNA-seq data [27,28]. RIN cut-offs between 5 and 8 have been suggested as one means of minimizing this risk [29–31]. However, depending on the research question, samples with lower quality can still generate useful data. In a study made on RNA extracted from brain glioblastoma cells, the authors found that differences in number of differentially expressed genes between RIN 10 and 8 were larger than between RIN 8 and 6. Therefore, they argue that there is no justification to include samples based on a specific RIN threshold. Instead they emphasized the importance of being mindful of the potential effects of low RIN values and recommended that all samples maintain comparable RIN values [32]. In a study of RNA extracted from buffy coat samples at five timepoints (degradation levels) the authors concluded that useful RNA sequencing data could be generated even from highly degraded samples, as long as the trait of interest was not associated with the distribution of RIN values. Also, including RIN values in the statistical model helped account for RNA degradation [28]. In our data, RNA integrity was correlated with the expression levels of housekeeping genes in the majority of the 23 genes assessed, with varying directions and magnitudes among the genes assessed and between the two library preparation methods. This is in line with earlier studies [27,28] and underscores the importance of consistent sample handling in comparison groups, such as disease cases and controls, in studies using RNA sequencing data, so as not to introduce batch effects related to RNA integrity. In our study, RIN value was positively correlated with number of uniquely mapped reads for poly-A data but no correlation was seen for Ribo-Zero. Other factors than the RIN value may also affect the expression profiles, e.g., possible sample contamination, batch effects related to sequencing runs and choice of alignment software.

A main strength of this investigation was the use of biobanked blood samples from population-based cohorts representing a wide range of storage times, from 9.8 to 22.9 years. RIN values were positively correlated with time in freezer, which was unexpected and might, speculatively, reflect variation in sample handling over time. The use of a training sample set followed by the NSHDS samples, both of which produced promising results in the RNA extraction stage, was also an advantage. Our samples are arguably of higher quality with respect to preanalytical factors than samples collected in most mature prospective cohorts, particularly with respect to collection-to-freezer time, which in our cohort was limited to one hour. However, this may also impact the generalizability of our results. The main limitation of our study was the relatively small sample size, limiting the exploratory analyses of differential gene expression between future CRC cases and matched control participants. This was an active decision on our part, reflecting the need for a pilot study before initiating analyses in a larger sample.

In conclusion, this study demonstrates the feasibility of using biobanked blood samples, collected in tubes with no RNA preservative and stored for up to 23 years, for sequencing of white blood cell RNA.

## Supporting information

S1 Table RIN and yield values for both NSHDS and training samples. RIN values were measured using the Eukaryote Total RNA Pico assay on the Agilent 2100 Bioanalyzer (Agilent Technologies). RNA yield is based on a sample eluate of 35 µ L. RNA concentration was measured using Qubit BR RNA assay (ThermoFisher Scientific) and measurements below the detection range (1 – 1000 ng/ µ L) were set as 0. For the four samples where core A and B were pooled before RNA sequencing, a new RIN value was measured on the pooled sample.
(XLSX)

S2 Fig Quality scores of raw reads in Ribo-Zero data, assessed with FastQC and MultiQC. (A). Total number of reads per sample. (B) Distribution of mean quality value across each base position. (C) Number of reads with average quality score. (D) GC distribution over all sequences.
(PDF)

S3 Fig Quality scores of raw reads in poly-A data, assessed with FastQC and MultiQC. (A). Total number of reads per sample. (B) Distribution of mean quality value across each base position. (C) Number of reads with average quality score. (D) GC distribution over all sequences.
(PDF)

S4 Fig Heatmap of Ribo-Zero data based on default clustering metric in R-function heatmap. Based on all genes that overlapped between Ribo-Zero and poly-A dataset after removing the 20% most low expressed genes.
(PDF)

S5 Fig Heatmap of poly-A data based on default clustering metric in R-function heatmap. Based on all genes that overlapped between Ribo-Zero and poly-A dataset after removing the 20% most low expressed genes.
(PDF)

S6 Table Differentially expressed genes with respect to sex for poly-A and Ribo-Zero data. (XLSX)

S7 Raw images Raw images from bioanalyzer related to Fig 2C and D and Fig 3B and D. Uncropped electropherogram and individual gel-image for each sample shown in Figure 2C-D and Fig 3B-D, as well as the complete gel-image for the whole bioanalyzer chip. Gel lanes with samples not included in Figs 2C-D and 3B-D are marked with an X.
(PDF)
