# Supplementary figures and images for: mRNA extracted from frozen buffy coat samples stored long term in tubes with no RNA preservative shows promise for downstream sequencing analyses
Source: PLoS One. 2025 Mar 19;20(3):e0318834. doi: 10.1371/journal.pone.0318834 (PMC11922291; doi:10.1371/journal.pone.0318834)

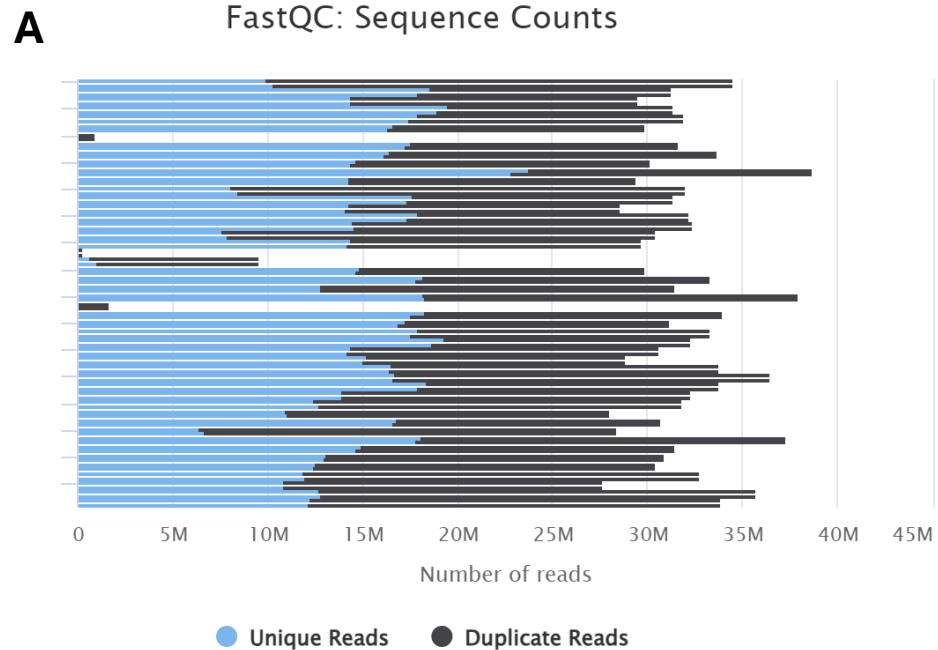

Created with MultiQC

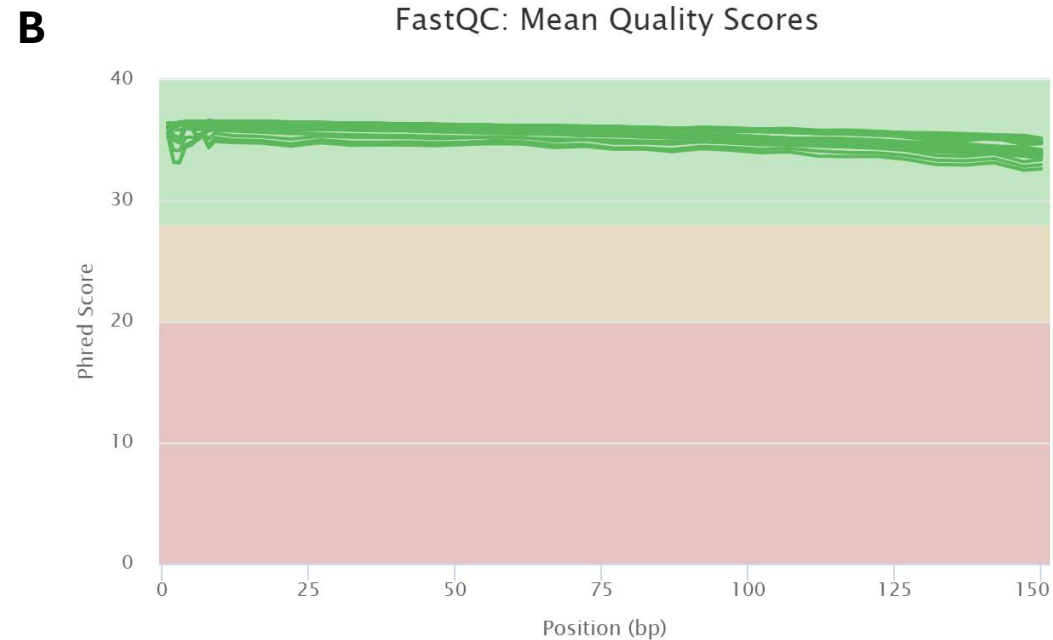

Created with MultiQC

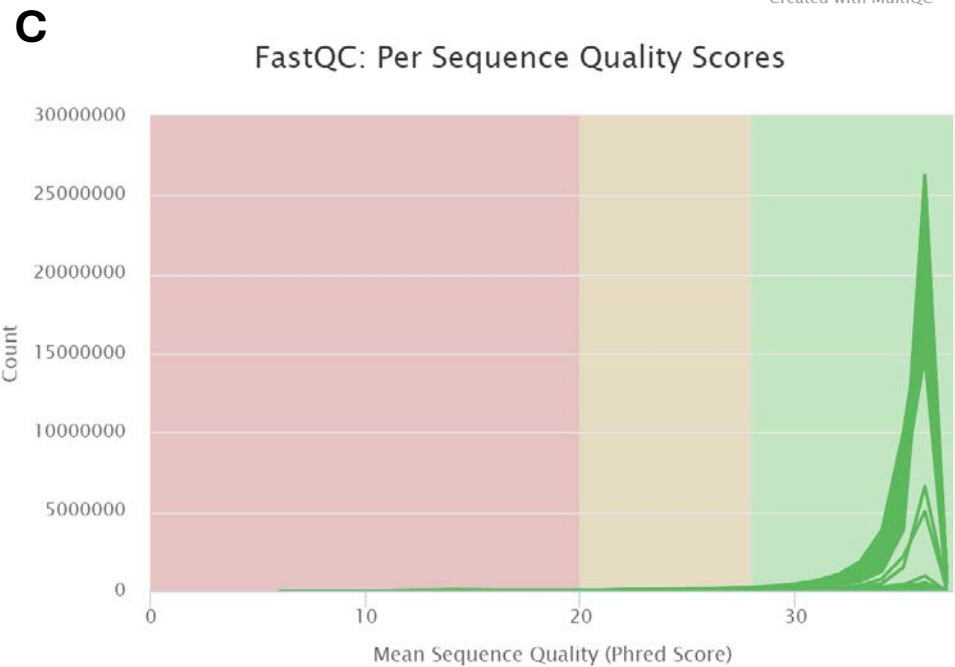

Created with MultiQC

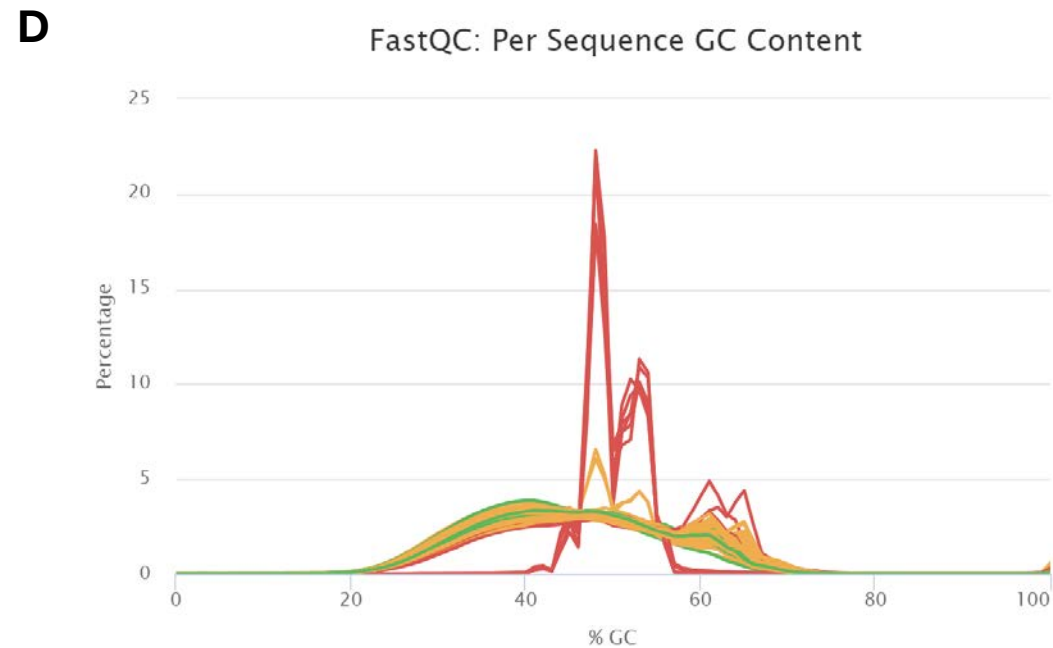

Created with MultiQC

Supplement: S2 Fig — (A). Total number of reads per sample. (B) Distribution of mean quality value across each base position. (C) Number of reads with average quality score. (D) GC distribution over all sequences. (PDF) [file pone.0318834.s002.pdf]

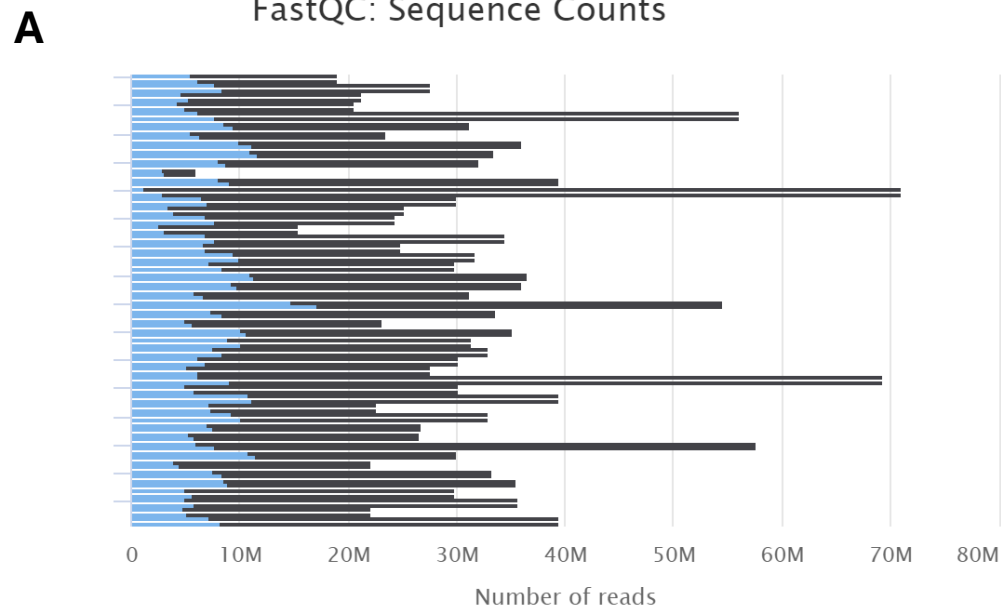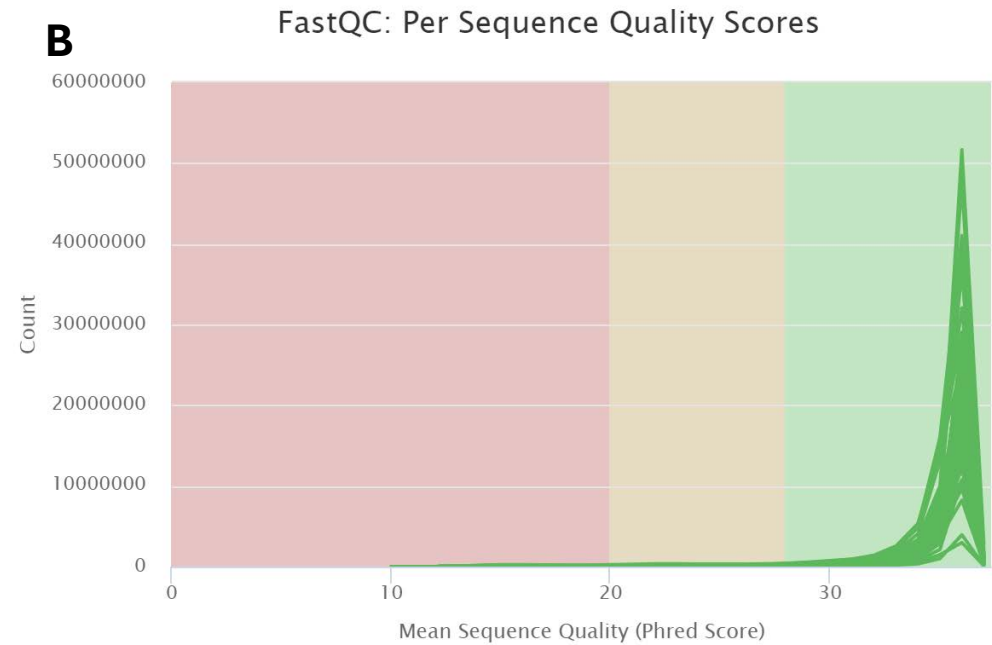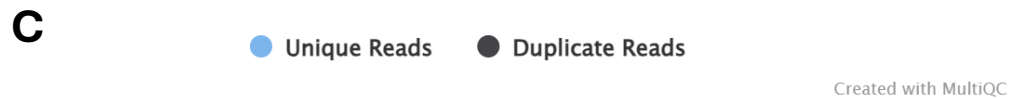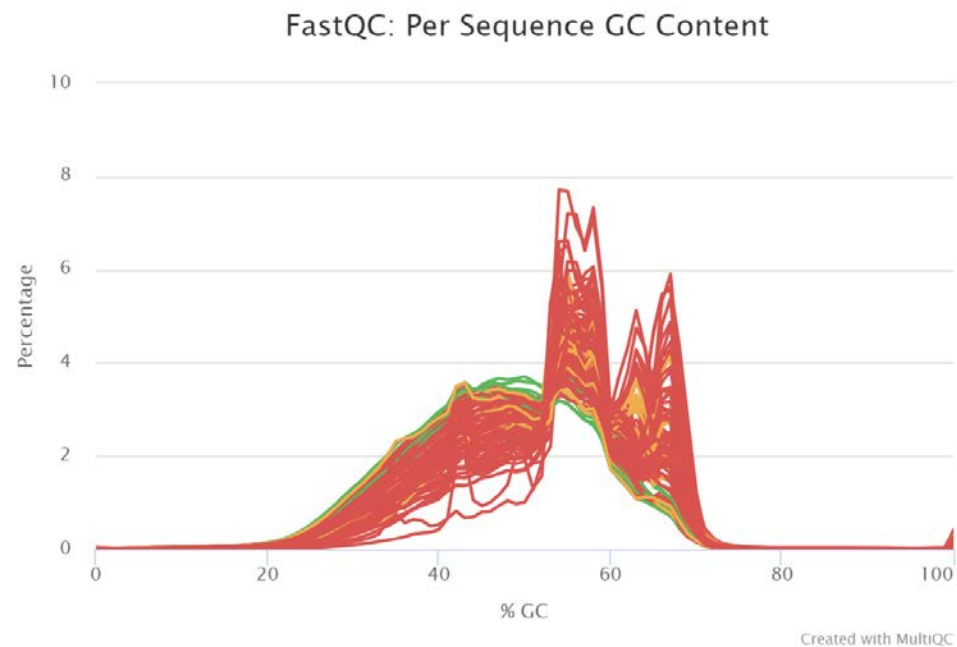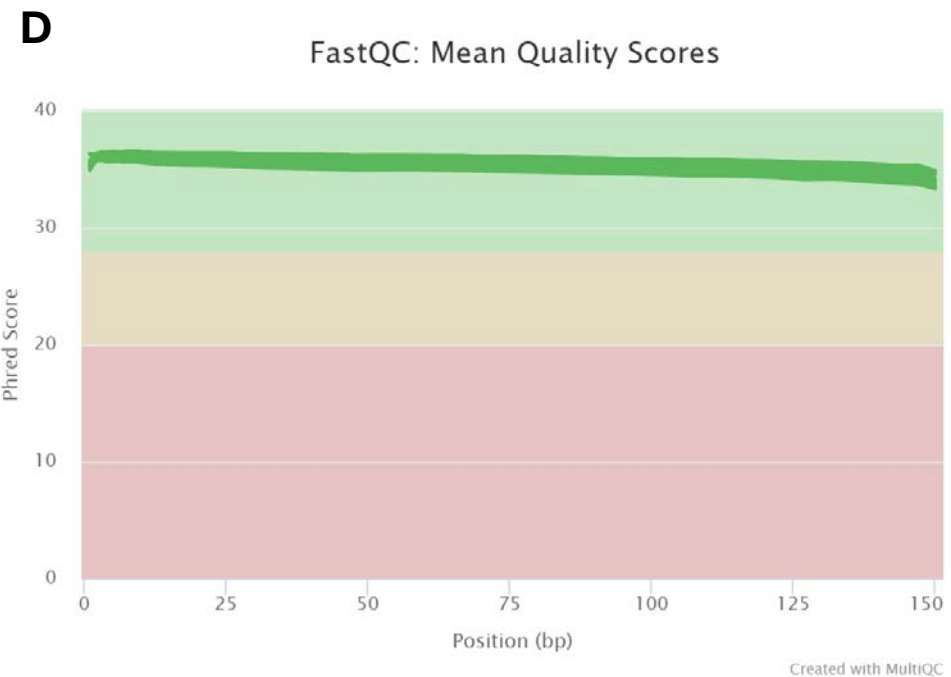

Supplement: S3 Fig — (A). Total number of reads per sample. (B) Distribution of mean quality value across each base position. (C) Number of reads with average quality score. (D) GC distribution over all sequences. (PDF) [file pone.0318834.s003.pdf]

# Ribo-Zero

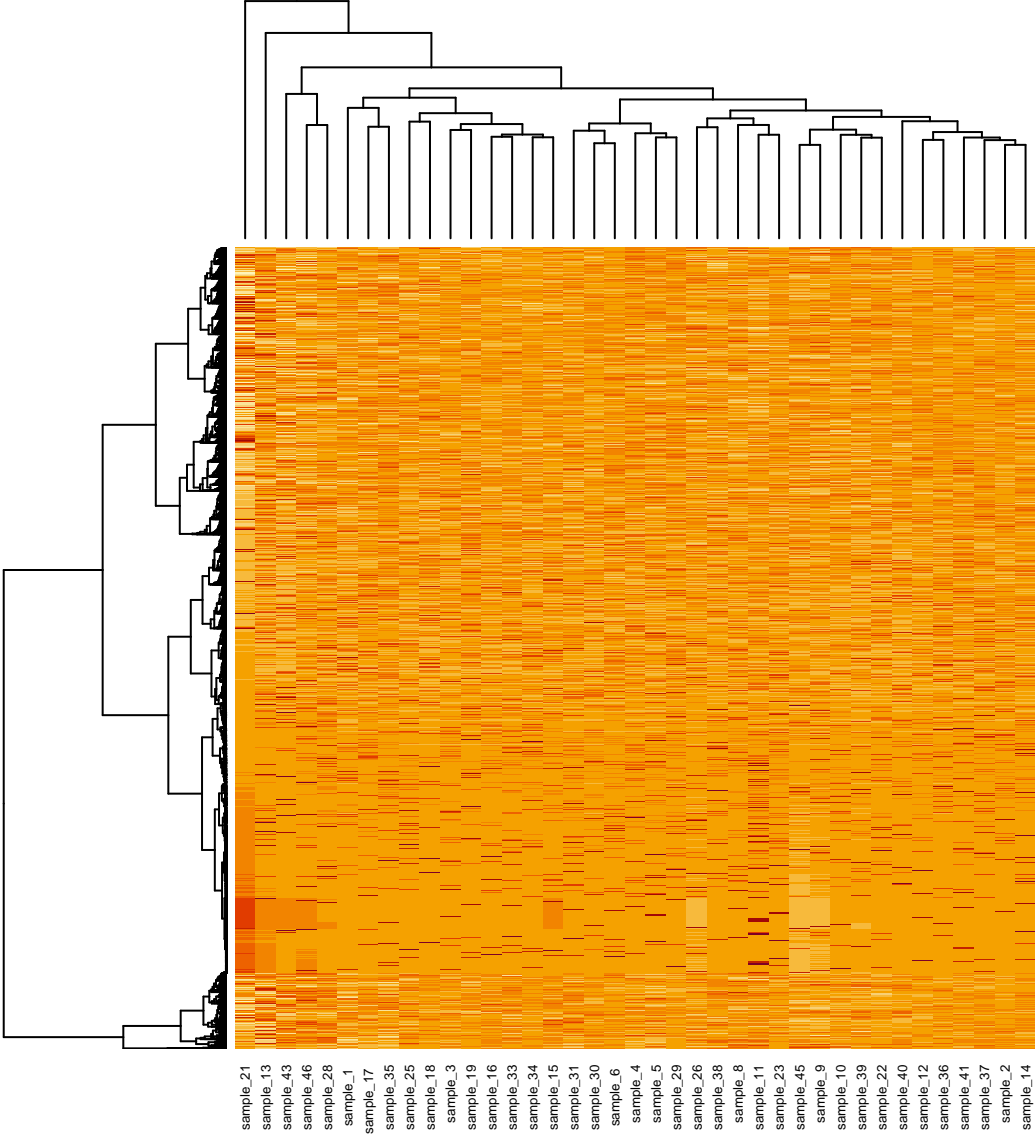

Supplement: S4 Fig — Based on all genes that overlapped between Ribo-Zero and poly-A dataset after removing the 20% most low expressed genes. (PDF) [file pone.0318834.s004.pdf]

# Poly-A

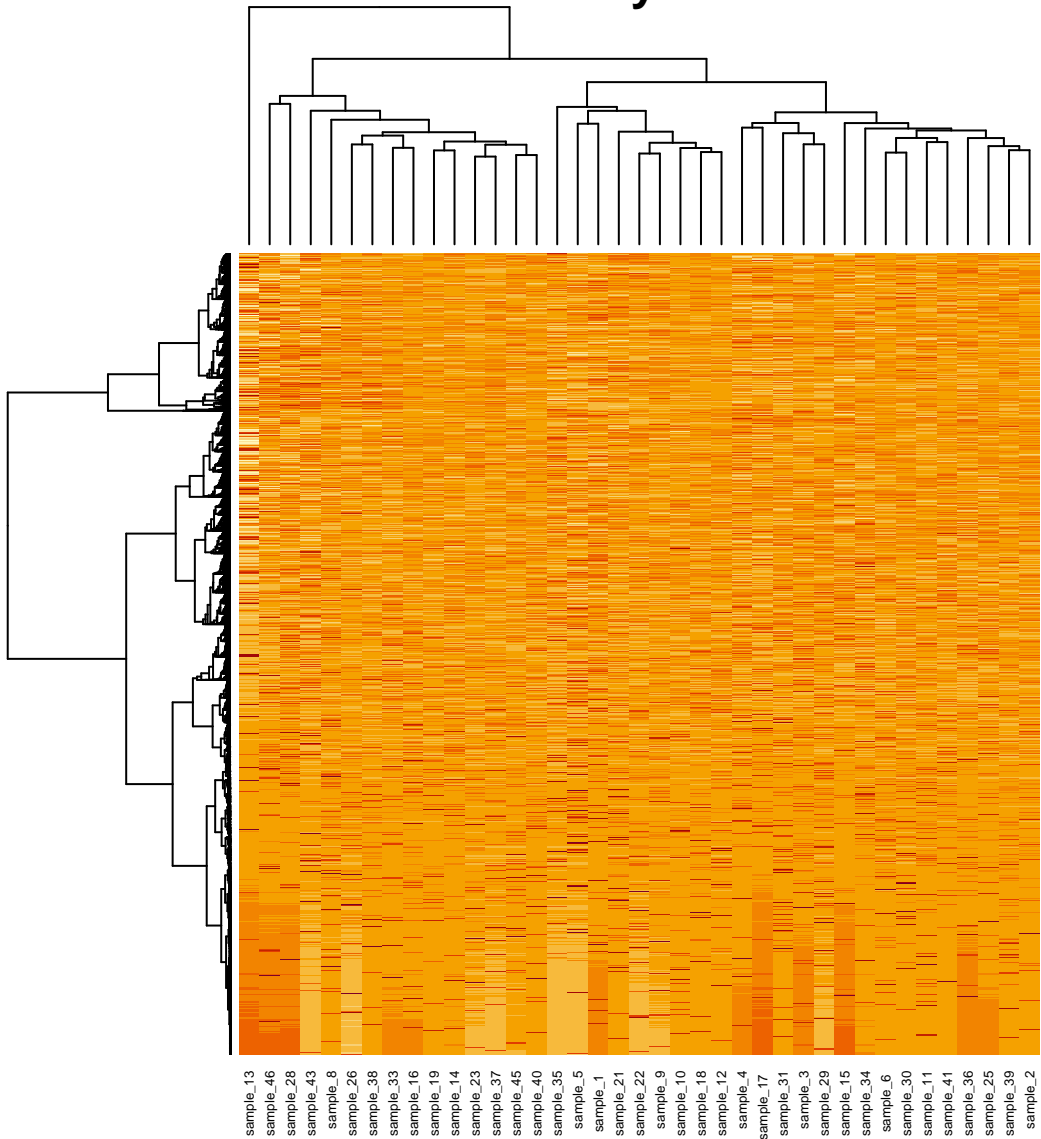

Supplement: S5 Fig — Based on all genes that overlapped between Ribo-Zero and poly-A dataset after removing the 20% most low expressed genes. (PDF) [file pone.0318834.s005.pdf]
