# Supplementary material for: mRNA extracted from frozen buffy coat samples stored long term in tubes with no RNA preservative shows promise for downstream sequencing analyses
Source: PLoS One. 2025 Mar 19;20(3):e0318834. doi: 10.1371/journal.pone.0318834 (PMC11922291; doi:10.1371/journal.pone.0318834)

Raw bioanalyzer images for Figure 2C

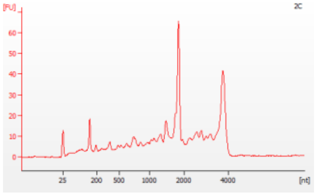

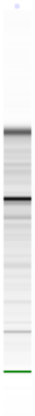

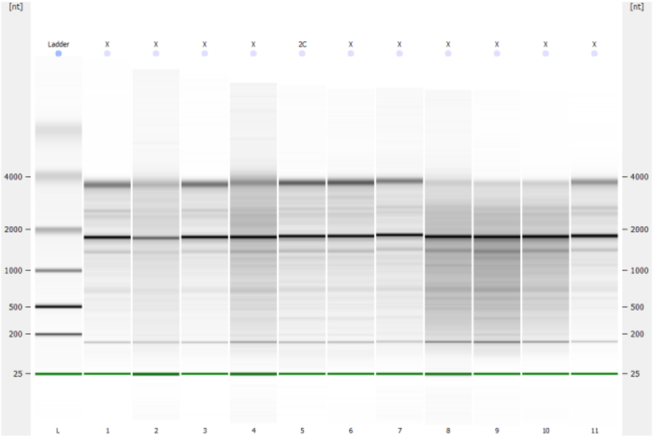

Raw bioanalyzer images for Figure 2D

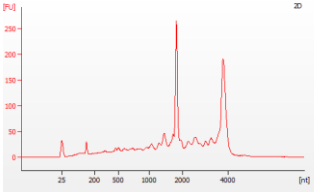

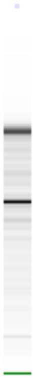

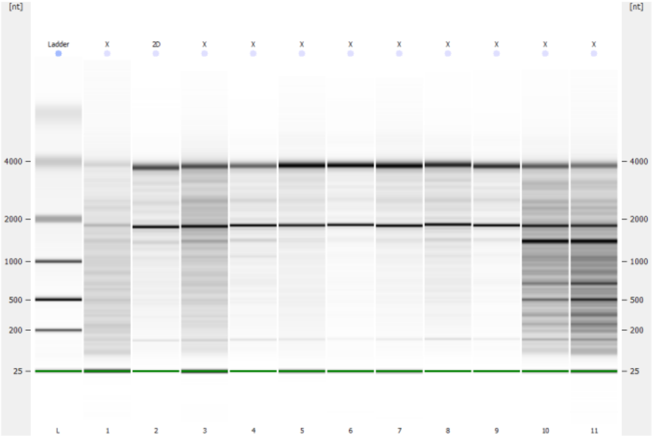

Raw bioanalyzer images for Figure 3B

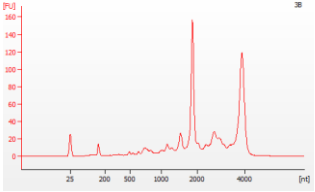

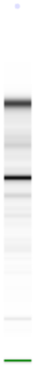

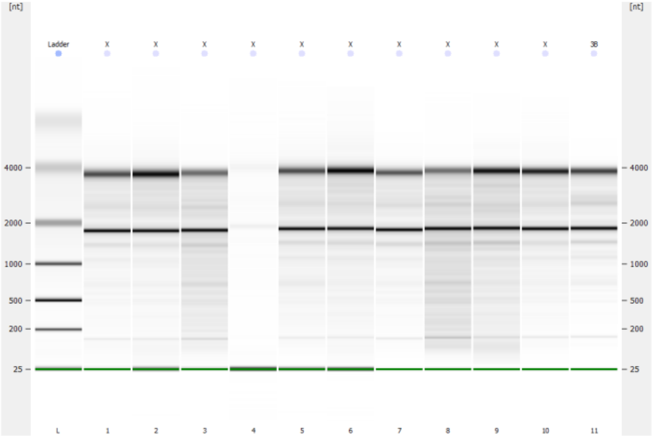

Raw bioanalyzer images for Figure 3C

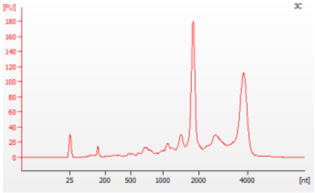

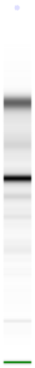

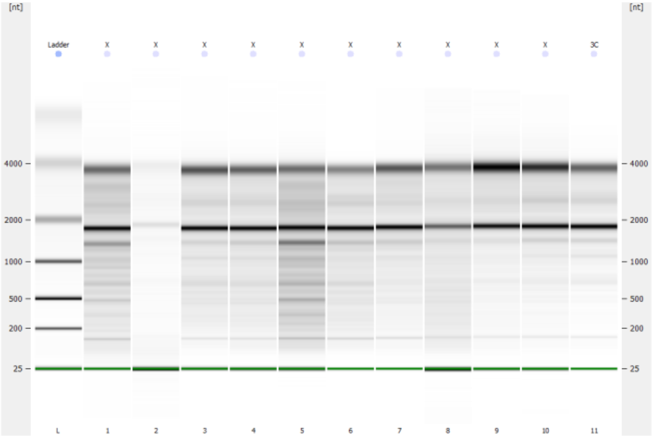

Raw bioanalyzer images for Figure 3D

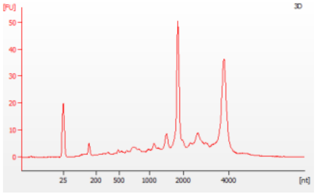

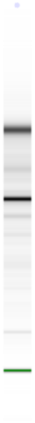

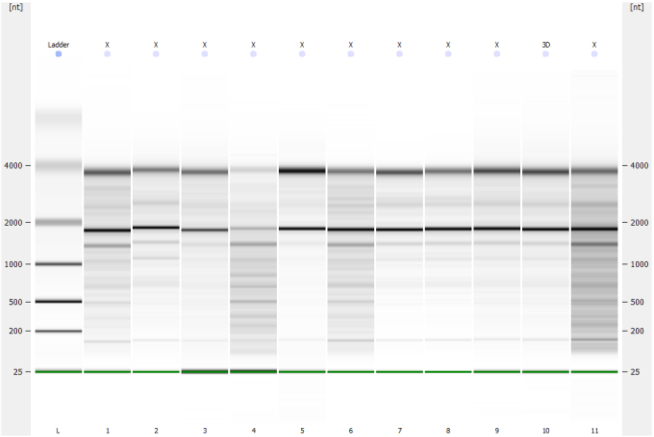

Supplement: S7 Raw images — Uncropped electropherogram and individual gel-image for each sample shown in Figure 2C-D and Fig 3B-D, as well as the complete gel-image for the whole bioanalyzer chip. Gel lanes with samples not included in Figs 2C-D and 3B-D are marked with an X. (PDF) [file pone.0318834.s007.pdf]
